# Trends, insights and effects of the Urban Wastewater Treatment Directive (91/271/EEC) implementation in the light of the Polish coastal zone eutrophication

**DOI:** 10.1007/s00267-020-01401-6

**Published:** 2021-01-15

**Authors:** Michał Preisner, Marzena Smol, Dominika Szołdrowska

**Affiliations:** 1 Mineral and Energy Economy Research Institute, Polish Academy of Sciences, Wybickiego Str. 7A, 31-261 Cracow, Poland; 2 AGH University of Science and Technology, al. Mickiewicza 30, 30-059 Cracow, Poland

**Keywords:** Urban wastewater treatment, Eutrophication, Baltic Sea, Nitrogen, Phosphorus, Nutrients

## Abstract

The intensification of the Baltic Sea eutrophication is associated with the increase of anthropogenic nutrients loads, mainly nitrogen and phosphorus introduced into surface waters from a diffuse, point and natural background sources. Despite the observed decreasing trends in nutrient concentrations in some parts of the Baltic Sea, eutrophication-related indicators continue to deteriorate. This accelerates harmful algal blooms and dissolved oxygen deficits resulting in severe ecosystem disturbance. The paper presents trends, insights and effects of the Urban Wastewater Treatment Directive 91/271/EEC implementation in Poland based on the nutrient riverine loads from Polish territory with particular attention given to the development of municipal wastewater treatment plants under the National Wastewater Treatment Programme 2003–2016. Environmental effects of wastewater infrastructure modernisation are investigated by using available data on the changing nutrient concentrations in the coastal water in 3 basins (Gdansk Basin, Bornholm Basin and Eastern Gotland Basin) belonging to the Polish Exclusive Economic Zone within the Baltic Sea. The results show that the decreasing trend regarding phosphorus loads reduction from municipal effluents was achieved while a stable trend with temporary increases was achieved in terms of nitrogen loads. Moreover, the investigation provides information about the potential bioavailability of discharged effluents before and after the Directive implementation by including total and inorganic forms of nitrogen and phosphorus in the analysis.

## Introduction

The Baltic Sea is one of the most severely polluted seas on the globe with eutrophication being its biggest threat resulting in harmful algal blooms (HABs) (Kahru et al. 2020) and dissolved oxygen (DO) deficits (Diaz and Rosenberg 2008; Breitburg et al. 2018). The intensification of eutrophication process is tightly associated with the increase of anthropogenic nutrients loads, mainly nitrogen (N) and phosphorus (P) introduced into surface waters from the point, diffuse and natural background sources (Kiedrzyńska et al. 2014; Neverova-Dziopak and Dan 2018). Depending on a specific water ecosystem conditions either N or P or both elements might be the limiting factor of eutrophication development (Granéli et al. 1990; Schindler 2006).

**Fig. 1:**
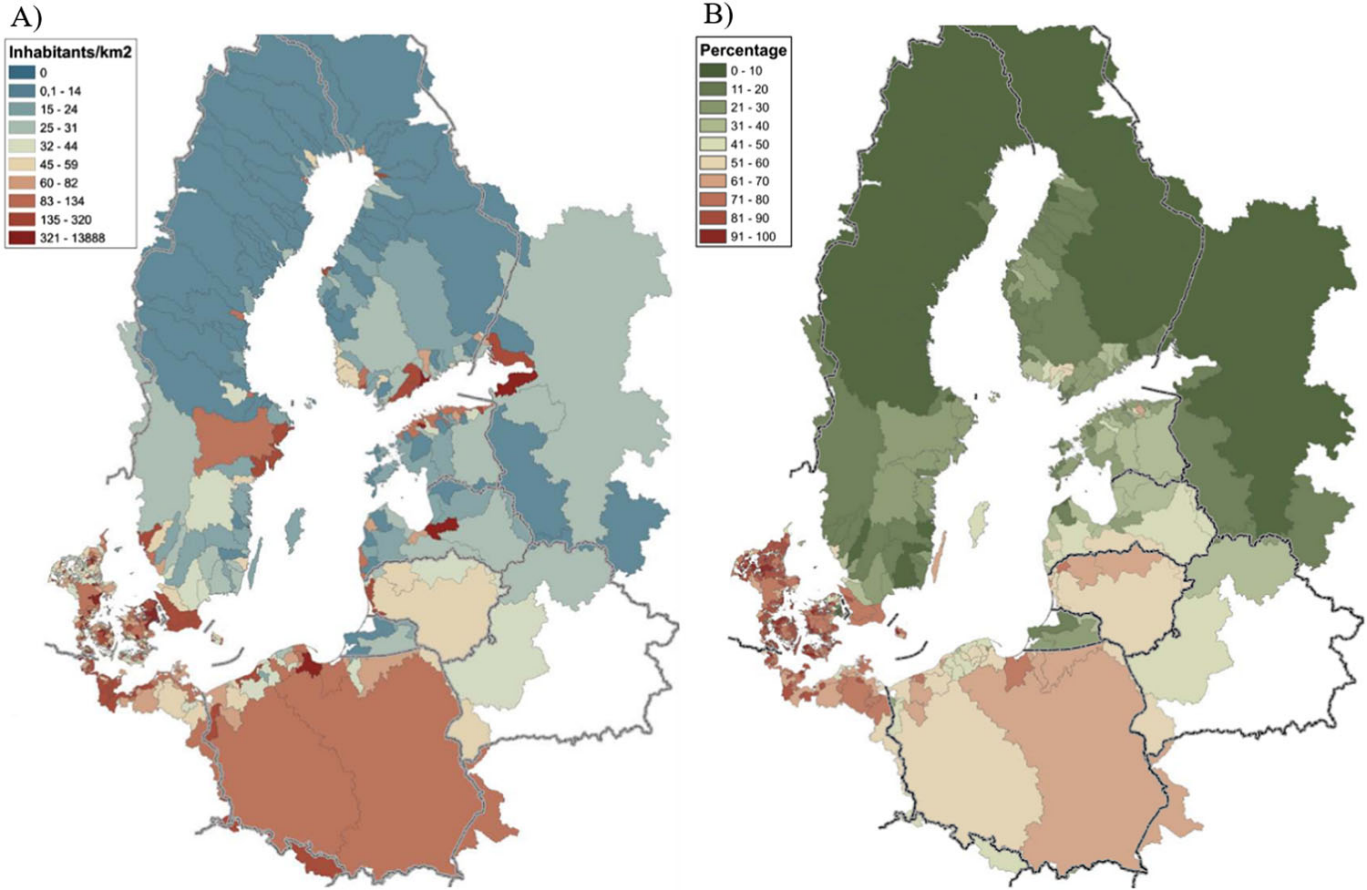
Inhabitants per km^2^ (A) and agricultural land share (B) in the Baltic Sea sub-catchments area (HELCOM 2018b)

The above issue was discovered in the 70–80s of the 20^th^ century, mainly due to excessive human activity in the Baltic Sea region which still becomes a major challenge (Raudsepp et al. 2019). Several regulations and strategies were developed to minimalize eutrophication in the European Union (EU) water environment including the Baltic Sea, e.g. the Urban Wastewater Treatment Directive 91/271/EEC (UWWTD), Nitrates Directive 91/676/EEC, Marine Strategy Framework Directive 2008/56/EC (MSFD), Water Framework Directive 2000/60/EC (WFD). Furthermore, the Helsinki Commission (HELCOM) have developed multiple measures and recommendations for all countries in the Baltic Sea region for efficient eutrophication mitigation. According to HELCOM data, Poland is the major nutrient polluter of the Baltic Sea with approx. 25% of total nitrogen (TN) and 32% of total phosphorus (TP) load (HELCOM 2018a). Such a state is mainly a result of the high urbanisation level and the share of agricultural land in Poland compering to other Baltic Sea countries as it is shown in Fig. 1.

To minimalize the anthropogenic eutrophication caused by excessive nutrient loads, HELCOM has proposed the nutrient input reduction targets in the Baltic Sea Action Plan (BSAP), which was adopted by all signatory countries of the Helsinki Convention in 2007 (Bring et al. 2015). The BSAP was revised in 2013 based on a new and more complete dataset as well as an improved modelling approach (Hasler et al. 2014). The revised BSAP sets the Maximum Allowable Inputs (MAI) and needed reductions for nutrients introduced to the Baltic Sea in which Poland was requested to reduce until 2021 its nutrients average annual load to approx. 153 600 Mg of TN and 4 400 Mg of TP (HELCOM 2011).

Unfortunately, as HELCOM assessed, although total nutrient loads were reduced, only 17 from 247 coastal and open water bodies achieved good status (HELCOM 2011). Moreover, none of the Baltic States achieved the nutrient MAIs in all waters particularly affected by eutrophication such as the Baltic Proper, Gulf of Finland and Gulf of Riga, while the nutrients inflow reduction was observed in waters where the restrictions are not obliged including the Danish Straits and Bothnian Sea (Svendsen et al. 2015). Moreover, despite that the nutrient riverine input has decreased by 12% of TN and by 25% of TP over the last decades, no significant improvement of the Baltic Sea state has been observed (HELCOM 2018a).

**Fig. 2:**
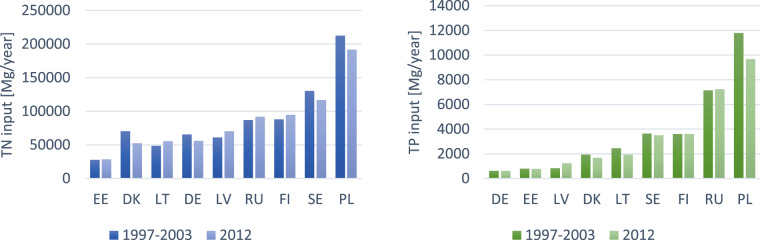
TN and TP inflow to the Baltic Sea by neighbouring countries (own diagrams, based on European Court of Auditors 2016)

The recent data on total nutrient inputs to the Baltic Sea show that Poland is the largest polluter of the Baltic Sea in terms of its trophic state. The nutrients loads discharged to the Baltic Sea from selected countries are shown in Fig. 2.

According to the European Court of Auditors (ECA), in the period 2003-2012, only Denmark, Poland and Sweden out of nine Baltic neighbouring countries achieved a downward trend for both nutrients (15.9%; 21.8%; 3.7% of P input reduction and 17.3%; 10.9%; 11.6% of N input reduction respectively) (European Court of Auditors 2016) while Latvia and Russia increased their share in general loads of both nutrients (33.3%; 1.2% of P input and 13.1%; 5.1% of N input respectively), whereas Finland increased only its TN load (by 7.1%). Germany managed to reduce only the TN load by 17.3% and Lithuania achieved TP load reduction by 27.7%. The nutrient load from Estonia has not significantly changed in the analysed period.

Therefore, it seems to be evident that Baltic Sea eutrophication remains a major environmental threat while the current situation highlights the need for more interdisciplinary research and integrated nutrient policy approach.

The current study presents trends, insights and effects of the UWWTD implementation in Poland based on the nutrient loads discharged from Polish territory with particular attention given to the development of municipal wastewater treatment plants (MWWTPs) and nutrient loads discharged with municipal effluents to the Baltic Sea. Environmental effects of wastewater infrastructure modernisation are investigated by using available data on the changing nutrient concentrations in the coastal water in the Polish Exclusive Economic Zone (EEZ) during 2007–2017.

## Materials and methods

The research focuses on the presentation of trends, insights and effects of the Urban Wastewater Treatment Directive 91/271/EEC implementation in Poland. Special attention was given to the discharged nutrients loads from MWWTPs and relevant parameters regarding the eutrophication process in the Polish coastal zone.

In the first step, the available publications in scientific databases such as Elsevier, ScienceDirect, Springer Link, MDPI database and Google Scholar were reviewed (Smol et al. 2020a). The most important legal documents and national statistics were analysed by desk-research including the following sources: Polish Central Statistical Office, EUR-Lex and Eurostat. The key documents in the research were reports prepared by the Polish General Inspectorate of Environmental Protection, National Water Management Holding—Polish Waters, Polish Institute of Meteorology and Water Management and the Helsinki Commission (HELCOM) on the condition of the Baltic Sea and inflowing pollutants. Particular attention was paid to the influent of N and P compounds including total and inorganic forms such as ammonia nitrogen (N-NH_4_), nitrate nitrogen (N-NO_3_), nitrite nitrogen (N-NO_2_) and orthophosphates (P-PO_4_). All literature items were screened based on the following keywords: “Urban wastewater treatment”, “Eutrophication”, “Baltic Sea”, “Nitrogen”, “Phosphorus”, “Nutrients”, “Municipal effluents”, “Nutrient input to the Baltic Sea”, “Waterborne N and P loads”, “Baltic Sea Action Plan”, “Gdansk Basin”, “Bornholm Basin”, “Eastern Gotland Basin”, “Coastal water pollution”.

**Fig. 3:**
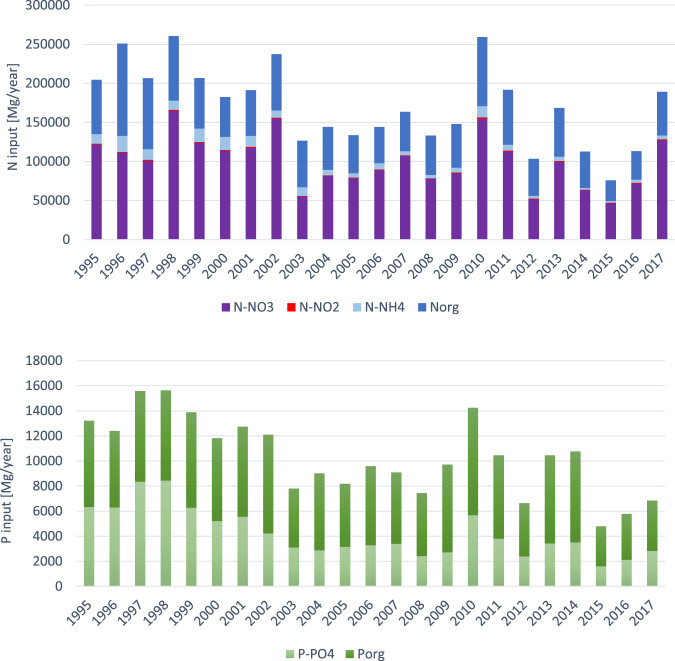
Riverine N and P input to the Baltic Sea from Polish territory (own diagrams, based on HELCOM 2019, 2018a)

The second step was to analyse and compile the available data, including the analysis of the waterborne loads of various N and P compounds from Polish territory in 1995–2017 (Fig. 3), number and the share of residents connected to the MWWTPs from urban and rural areas (Table 1) and identification of main sources of excessive nutrient loads introduced to the Baltic Sea.

**Table 1:** Number and share of inhabitants connected to MWWTPs according to their types (NWMH Polish Waters 2018; Polish Central Statistical Office 2020)

	1995	2000	2005	2011	2014	2015	2016
	**Inhabitants connected to MWWTPs**						
Urban areas	15,554,500	18,928,100	19,955,400	20,670,900	21,791,600	21,906,300	21,932,900
Inhabitants share	65.7%	80.0%	85.2%	88.4%	94.0%	94.6%	94.8%
Rural areas	450,300	1,576,400	3,005,500	4,631,100	5,702,000	6,049,400	6,312,900
Inhabitants share	3.1%	10.8%	20.4%	30.6%	37.4%	40.0%	41.3%
	**Mechanical and mechanical-chemical MWWTPs**						
Urban areas	2,947,200	1,271,800	750,000	17,700	9000	9000	8900
	**Biological and mechanical-biological MWWTPs**						
Urban areas	11,073,400	10,290,500	6,115,000	2,623,000	2,295,100	2,195,300	2,127,400
	**Enhanced biological nutrient removal (EBNR) MWWTPs**						
Urban areas	1,110,200	7,329,000	13,090,400	18,030,200	19,487,400	19,701,800	19,796,600

The method used for the assessment of the National Municipal Wastewater Treatment Programme (NMWTP) implementation in Poland was based on the data analysis concerning nutrient discharges present in the annual reports from Polish MWWTPs submitted to the national water and wastewater authority—National Water Management Holding—Polish Waters. The data included in the analysis consisted of nutrient content in wastewater from a total of 1934 MWWTPs present in the most recent available report from 2017 (Fig. 5). The analysed period regarding the riverine nutrient loads discharged to the Polish Baltic Sea coastal zone covered the years 1995 – 2017 in which loads of total and inorganic N and P were analysed, while the share of dissolved inorganic nitrogen (DIN) and dissolved inorganic phosphorus (DIP) in their total content were calculated according to Eqs. 1 and 2 respectively.

1 $${\mathrm{DIN}}\, {\rm{share}} = \frac{{({\mathrm{N}} - {\mathrm{NO}}_3) + ({\mathrm{N}} - {\mathrm{NO}}_2) + ({\mathrm{N}} - {\mathrm{NH}}_4)}}{{{\mathrm{TN}}}}[{\mathrm{\% }}]$$

2 $${\mathrm{DIP}}\, {\rm{share}} = \frac{{{\mathrm{P}} - {\mathrm{PO}}_4}}{{{\mathrm{TP}}}}\left[ {\mathrm{\% }} \right]$$

**Fig. 4:**
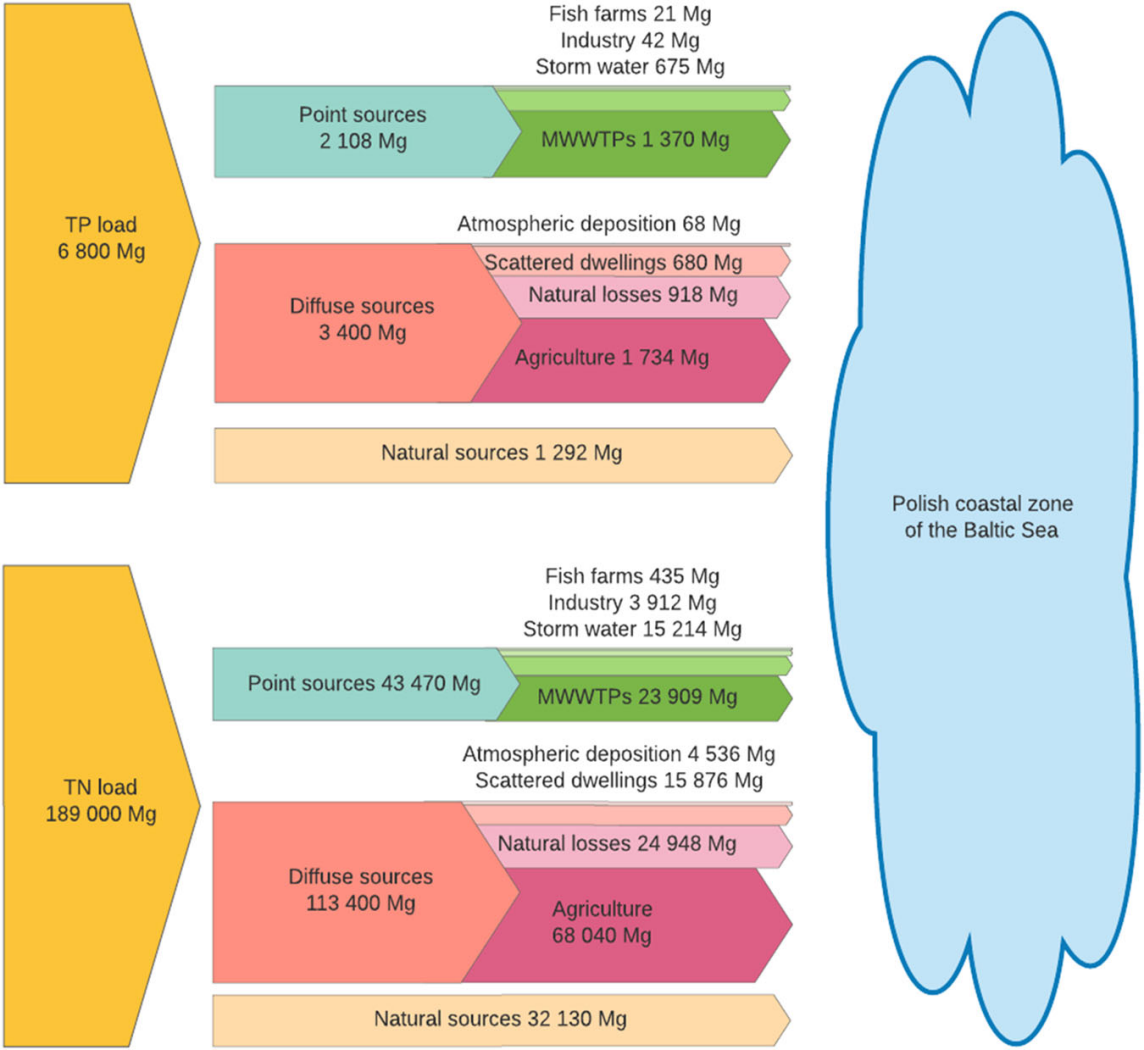
Sources of nutrients introduced to the Baltic Sea from Poland in 2017 (own diagram, based on HELCOM 2018b)

Furthermore, based on the riverine annual nutrient export obtained from HELCOM compilations of pollution load data (HELCOM 2018c) and its share regarding various sources and pathways (HELCOM 2018b) the mass flow of discharged nutrients to the Polish Baltic Sea coastal zone was developed (Fig. 4).

The study was focused on the south-eastern part of the Baltic Sea in the Polish EZZ which for the purpose of this study was divided into the following basins: Gdansk Basin, Bornholm Basin and Eastern Gotland Basin in order to assess the effectiveness of measures to mitigate the effects of eutrophication. The nutrient concentration data used in the study were obtained from the national monitoring system managed by the Polish General Inspectorate of Environmental Protection which during 2007 and 2017 have monitored the Polish EEZ in terms of nutrient content (total and dissolved forms), chlorophyll *a*, dissolved oxygen concentration and water transparency. For this study, data from 11 monitoring points were used (Fig. 6).

## Results

This section provides an overview of the results regarding nutrient inputs to the Baltic Sea from the Polish territory in the light of measures taken in Poland to mitigate eutrophication of the Baltic Sea. Moreover, insights regarding the implementation of the UWWTD in Poland are presented concerning the observed trends in water quality of 3 basins analysed in the Polish coastal zone in terms of N and P concentrations.

### Polish contribution to the Baltic Sea nutrient pollution

The analysis of the riverine loads of TN and TP from Polish territory shows a decreasing trend in 1995–2017. The riverine organic and inorganic nutrient input from Poland to the Baltic Sea, including transboundary loads is shown in Fig. 3 (HELCOM 2018a, 2019). The transboundary loads were estimated by HELCOM to reach approx. 5% in terms of TN and 4.3% regarding TP (HELCOM 2018b).

Many EU activities are aimed at the protection of the Baltic Sea, not only in terms of legal regulations but also economic support (Smol et al. 2020b). One of the most important tools supporting the abatement of eutrophication in the region is the European Regional Development Fund (ERDF), from which multiple infrastructure investments in wastewater sector were co-financed in the Member States in the Baltic Sea region (Avdiushchenko 2018). The ERDF support for Poland reached 3.4 billion Euro out of a total of 4.6 billion Euro during 2007–2013 (European Court of Auditors 2016). Moreover, due to the EU’s financial support, it was possible to modernise 1575 municipal wastewater treatment plants (MWWTPs) and build 403 new MWWTPs (Preisner 2020). All of the investments in the wastewater infrastructure were estimated at approx. 15 billion Euro carried out under the NMWTP during 2003-2016 which was a response to the UWWTD requirements and its transposition in Poland. The results of the NMWTP implementation in Poland are presented in Table 1.

Due to the NMWTP implementation, it was possible to provide access to the municipal sewage system to approx. 95% of urban areas inhabitants and 41.3% of rural areas inhabitants in 2016 while in 2005 there were approx. 85.2% and 20.4% of residents connected to the sewage system in urban and rural areas respectively. Moreover, the number of inhabitants connected to MWWTPs with EBNR has increased approx. from 1.1 million in 1995 to 13.1 million in 2005 and finally to 19.8 million in 2016 (NWMH Polish Waters 2018).

### Bioavailability aspect of the discharged nutrient loads

The content and share of inorganic N and P compounds are important measures of the eutrophication potential related to the nutrient loads. Inorganic nutrient compounds are considered as the most bioavailable for aquatic vegetation, which was shown by various studies (Nakajima et al. 2006; Li and Brett 2015). Inorganic P form as P-PO_4_ is directly absorbed by plankton and higher aquatic vegetation (Boström et al. 1988). Phytoplankton species that easily absorb P-PO_4_ include *Anabaena flos-aque*, *Selenastrum capricornutum* and *Cladophora sp*. (Sharpley et al. 1994; Warwick et al. 2013; Venkiteshwaran et al. 2018). The content of P individual forms in water environment depends on the pH value: at a water pH of 3-7 there are usually present as H_2_PO_4_ forms, at a pH of 8-12 as HPO_4_ forms (Schindler 2006). Planktonic organisms assimilate inorganic nitrogen and some organic nitrogen (N_org_) compounds. Inorganic nitrogen forms, such as N-NO_3_ and N-NH_4_ predominate in surface waters (Neverova-Dziopak 2010). N-NO_3_ can be easily absorbed by Cyanobacteria species (e.g. *Anabaena variabilis, Nostoc paludosum* and *Nostoc coeruleum*) (Zaragüeta and Acebes 2017). Furthermore, some algae species reproduce better by absorbing N-NH_4_ e.g. *Anabaena Microcysatis aeruginosa, Calothrix elenkini, Hapalosiphon fomtinalis* and others (Zeng et al. 2014).

The selected species of aquatic vegetation (e.g. Cyanobacteria) are able to compensate N in the absence of its availability in the water environment by binding molecular N from the atmosphere (Wall 2013). In turn, the least bioavailable mineral N form is N-NO_2_, which is a transitional product of the biological decomposition of nitrogen-containing organic compounds and occurs in surface waters in negligible amounts (Wang et al. 2019). Almost every form of N present in waters can stimulate the eutrophication process and lead to an increase in biological productivity of waters (Howarth and Marino 2006). Temperature, oxygen conditions and biological processes (e.g. N fixation ratio) can affect the dominant N forms occurring in a given aquatic environment (Golubkov et al. 2019).

The trends in the share of DIN and DIP in total nutrient content were analysed in the years before (1995–2003) and after (2004–2017) the beginning of the NMWTP implementation. In the analysed period the following trends regarding the content of inorganic nutrient fractions were observed: a decreasing trend of inorganic P share in TP (average 45% in 1995–2003 and 35% in 2004–2017) and a stable share of inorganic N (average 64% in 1995–2003 and also in 2004–2017) as presented in Table 2.

**Table 2:** The share of inorganics in total N and P content in the riverine nutrients loads during 1995–2017 in Poland

Year	TN load	Share of DIN in TN	Average	TP load	Share of DIP in TP	Average	Riverine average flow (m^3^/s)
1995	204,674	66%	Average TN load before the NMWTP implementation = 207,444 Mg Average share of DIN in TN before the NMWTP implementation = 64%	13,208	48%	Average TP load before the NMWTP implementation = 12,797 Mg Average share of DIP in TP before the NMWTP implementation = 46%	1744
1996	250,876	53%		12,389	51%		1888
1997	206,720	56%		15,580	54%		2062
1998	260,470	68%		15,646	54%		2257
1999	206,840	69%		13,880	45%		2193
2000	182,325	72%		11,821	44%		1978
2001	191,242	70%		12,746	44%		2062
2002	237,359	70%		12,094	35%		2146
2003	126,487	53%		7805	40%		1357
2004	144,082	62%	Average TN load after the NMWTP implementation = 148 607 Mg Average share of DIN in TN after the NMWTP implementation = 64%	9016	32%	Average TP load after the NMWTP implementation = 8 783 Mg Average share of DIP in TP after the NMWTP implementation = 35%	1395
2005	133,615	64%		8181	39%		1492
2006	144,074	68%		9587	34%		1550
2007	163,621	69%		9087	37%		1645
2008	133,182	62%		7443	33%		1422
2009	147,831	62%		9718	28%		1635
2010	259,190	66%		14,244	40%		2744
2011	191,838	63%		10,443	36%		1995
2012	103,368	54%		6645	36%		1407
2013	168,660	63%		10,446	33%		1752
2014	112,767	59%		10,761	33%		1084
2015	75,908	65%		4785	34%		1109
2016	113,142	68%		5773	37%		1271
2017	189,219	70%		6838	41%		No data

It needs to be mentioned that the peak values observed in 2010 for both N and P loads and the share of inorganic compounds most probably results from a severe flood that took place in eastern Europe. Intensive rains can explain the higher nutrient loads introduced by Polish rivers to the Baltic sea while surface flow affects nutrient inputs from natural and agricultural sources (Nyenje et al. 2010; Thieu et al. 2010; Kordana and Słyś 2020).

Unfortunately, despite the investments in wastewater treatment infrastructure by the NMWTP implementation nearly 90% rivers, 75% lakes and 100% coastal and transitional waters in Poland are still threatened by the risk of eutrophication (Wojciechowska et al. 2019). This confirms a high inefficiency of the actions aimed at eutrophication mitigation in Poland and highlights that a major revision is needed in all legal and technical measures to allow for more cost-efficient eutrophication control (Miłaszewski 2018; Preisner et al. 2020a).

### Identification of main sources of nutrient loads introduced to the Baltic Sea from Polish territory

The identified sources of nutrients introduced to the Baltic Sea are divided into a point, diffuse and natural background sources. The detailed information on the nutrient sources in the Polish Baltic Sea water catchment is presented in Fig. 4. The point sources of nutrients are responsible for 31% of TP and 23% of TN introduced to the Baltic Sea, while the natural background is responsible for 19% of TP and 17% of TN in total load input (HELCOM 2018a). The diffuse sources are mainly responsible for nutrients loads introduced to the Baltic Sea from Polish catchment, and they stand for 60 and 50% of the total load of TN and TP, respectively (HELCOM 2018d). Considering the point sources in detail, it should be noted that for both TP and TN, the largest contribution is related to MWWTPs (65% for TP and 55% for TN), secondly to storm waters (32% for TP and 35% for TN), industry (9% for TP and 2% for TN) and fish farms (1% for TP and 1% for TN). The MWWTPs discharges together with storm waters are responsible for 97% of TP and 90% of TN load from point sources (HELCOM 2019). On the other hand, agriculture is the main diffuse nutrient source, contributing to 51% of TP and 60% of TN load from all diffuse sources (HELCOM 2018a; McCrackin et al. 2018). Based on the known riverine annual nutrient export and its share regarding various sources the mass flow of discharged nutrient to the Baltic Sea from Polish territory in 2017 is shown in Fig. 4.

### Municipal wastewater nutrients input

**Fig. 5:**
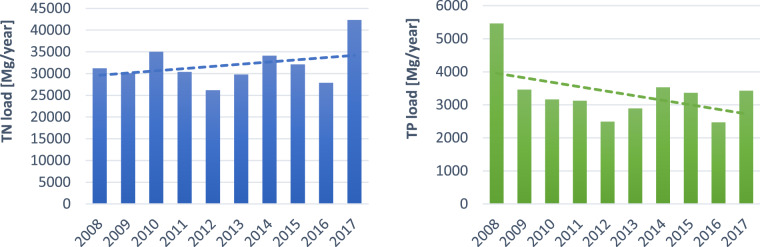
Annual average TN and TP load from Polish MWWTPs (own diagrams, based on Polish Waters 2017)

Municipal wastewater discharges have a great impact on the water trophic state due to their high content of N and P compounds (Li et al. 2012; Neverova-Dziopak and Kowalewski 2014; Gillmore et al. 2016). Municipal effluents loads are constantly increasing along with the population growth and the development of urbanisation processes (Lin et al. 2020). In connection with the above, the main direction in the prevention of eutrophication has become the reduction of nutrients introduced into wastewater receivers (Li and Brett 2012). The basic tool for achieving this goal were the legal requirements developed practically in all countries regarding the conditions for wastewater discharge into receiving waters, which are systematically becoming stricter in terms of nutrient content (Bohman 2018; Preisner et al. 2020b). This forced the use of advanced technologies for their removal in complex and expensive wastewater treatment systems (Surratt and Aumen 2014; Smol et al. 2018). It is a well-known fact that mainly the dissolved inorganic forms of nutrients are directly available to aquatic vegetation, while in rare cases, also some organic compounds (Czerwionka 2016). Municipal wastewater is rich in these bioavailable nutrient forms and determines the development of phytoplankton and macrophytes (Li 2014; Jin et al. 2019). The annual average of the TN and TP loads based on the reports from Polish MWWTPs are presented in Fig. 5.

There are two different trends observed for TP and TN. Regarding TP loads it seems that high financial investments in wastewater infrastructure co-financed by the EU resulted in the reduction of TP load discharged to wastewater receivers and ending in the Baltic Sea. TP load from MWWTPs was the highest at the beginning of the analysed period in 2008 (5462 Mg/year), when infrastructural programmes were under implementation. The EBNR technology with optional chemical P precipitation introduced in many MWWTPs allowed to achieve in 2016 a TP load of 2473 Mg/year. Efforts to reduce the TN load were less satisfactory, however, this might be a result of the increasing amount of wastewater treated in MWWTPs. Therefore, TN shows an increasing trend with a peak value in 2017 (42,307 Mg/year). The lowest TN loads were observed in 2012 (2490 Mg/year) and 2016 (2473 Mg/year). It is important to mention that at the beginning of the NMWTP implementation in Poland many inhabitants were not connected to MWWTPs. For example in 2000 wastewater from only 80% of inhabitants in urban areas and only 10.8% of rural areas inhabitants was transported by the sewage system to MWWTPs. In the rural areas, most households were using septic tanks which were rarely leakproof while poorer users were trying to save expenses on sewage collection frequently causing surface and groundwater pollution.

### Polish coastal zone eutrophication

**Fig. 6:**
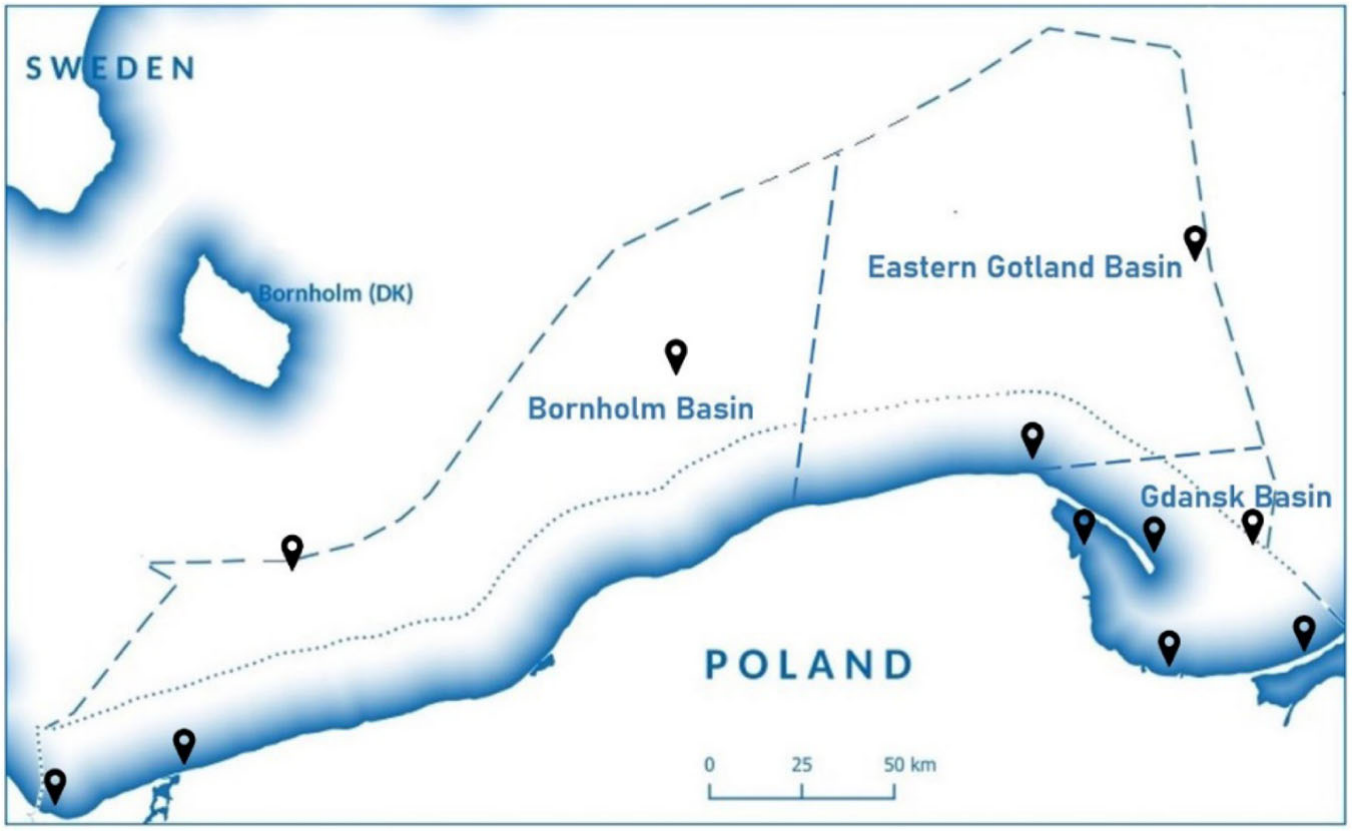
Location of the analysed basins with monitoring points in the Polish EEZ on the Baltic Sea (own diagram, based on General Inspectorate of Environmental Protection 2018)

The riverine nutrients loads from Polish territory are discharged to the part of the Baltic Sea so-called the Polish EEZ which is divided into 3 basins to provide more efficient monitoring of water pollution. The basins belonging to the EEZ are the Gdansk Basin, Bornholm Basin and Eastern Gotland Basin as presented in Fig. 6. According to HELCOM recommendations, the nutrient content, chlorophyll *a*, dissolved oxygen concentration and water transparency are monitored in the 3 basins by the Polish General Inspectorate of Environmental Protection.

The eutrophication holistic assessment has proceeded following the Marine Strategy Framework Directive 2008/56/EC (MSFD) guidelines for the separated basins in 2017. For the assessment key factors were used which contribute to the achievement of “good environmental status” (GES) which according to the MSFD is such state of the marine waters environment, ensuring ecologically diverse and dynamic oceans and seas, which are clean, healthy and fertile concerning their conditions, and the use of the marine environment occurs at a level that is sustainable and guarantees the preservation of the possibility to be used by present and future generations (EC 2008). Unfortunately, in 2017 none of the assessed indicators has reached the GES value and consequently, the examined basins in the Polish EEZ have not achieved “good” status (General Inspectorate of Environmental Protection 2018). In terms of eutrophication, the environmental state of Polish coastal waters in 2017 was assumed as inadequate concerning the MSFD requirements. The reason for such status are mainly excessive concentrations of N and P (in terms of mineral and organic forms), dissolved oxygen deficits and HABs occurrence what consequently resulted in the exceedance in chlorophyll *a* concentrations in all of the examined basins in the Polish EEZ.

### Eutrophication mitigation effects evidence and trends

To assess the effectiveness of eutrophication mitigation affords taken in the Polish EEZ within the NMWTP as the UWWTD transposition, the available monitoring data on total and inorganic nutrient compounds were analysed. The DIN and DIP content was measured by the Polish General Inspectorate of Environmental Protection mainly in winter, when according to the natural seasonal cycle, primary production disappears and the concentration of biogenic compounds is the highest during the year (Rogowska et al. 2019). According to the Eutrophication Assessment Manual (EAM) provided by HELCOM December, January and February are treated as winter months (HELCOM 2015). The total and inorganic nutrient content during 2007–2017 in the analysed basins is shown in Tables 3 and 4.

**Table 3:** Total and inorganic N content during 2007–2017 in the analysed basins in the Polish EEZ (own table, based on (General Inspectorate of Environmental Protection 2018)

Year	Gdansk Basin		Bornholm Basin		Eastern Gotland Basin	
	TN [mmol/m^3^]	DIN [mmol/m^3^]	TN [mmol/m^3^]	DIN [mmol/m^3^]	TN [mmol/m^3^]	DIN [mmol/m^3^]
2007	22.50	6.60	23.50	7.60	19.20	4.00
2008	24.50	6.00	23.80	2.90	20.80	2.50
2009	25.50	4.60	25.20	5.00	24.10	3.40
2010	29.00	9.30	29.20	6.90	24.00	4.60
2011	26.90	7.80	27.90	6.60	23.80	9.80
2012	22.40	2.60	22.50	6.10	21.90	3.20
2013	30.70	5.20	29.60	5.80	24.30	2.70
2014	28.20	4.10	25.50	4.80	25.80	4.30
2015	27.90	5.90	27.50	5.60	25.10	4.60
2016	29.10	5.70	25.10	3.20	24.80	2.90
2017	28.70	9.50	28.20	9.30	27.70	4.90

**Table 4:** Total and inorganic P content during 2007–2017 in the analysed basins in the Polish EEZ (own table, based on (General Inspectorate of Environmental Protection 2018)

Year	Gdansk Basin		Bornholm Basin		Eastern Gotland Basin	
	TN [mmol/m^3^]	DIN [mmol/m^3^]	TN [mmol/m^3^]	DIN [mmol/m^3^]	TN [mmol/m^3^]	DIN [mmol/m^3^]
2007	0.70	0.61	0.75	0.72	0.66	0.58
2008	0.72	0.59	0.74	0.56	0.70	0.50
2009	0.82	0.52	0.92	0.55	0.70	0.56
2010	0.98	0.76	0.90	0.63	0.85	0.68
2011	0.99	0.46	0.88	0.33	0.95	0.39
2012	0.88	0.38	0.96	0.43	0.90	0.33
2013	0.82	0.28	0.81	0.32	0.67	0.34
2014	0.72	0.43	0.80	0.37	0.88	0.45
2015	0.78	0.70	0.85	0.61	0.88	0.70
2016	0.68	0.82	0.66	0.79	0.59	0.72
2017	1.43	0.70	1.47	0.73	1.28	0.68

The TN content increasing trend was observed in all three analysed basins, however, in the Bornholm Basin, it was the most moderate what can be indirectly affected by the North Sea water inflows (Thieu et al. 2010; Hong et al. 2012). The lowest content of TN in all three basins was noticed in 2007 and 2012, while the highest values were achieved in 2013 in the Gdansk and Bornholm Basin and 2014 in the Eastern Gotland Basin.

During the analysed period there has been a rising trend identified in TP content in all of the analysed basins, which is partly a result of the peak reached in 2017. The TP content had an increasing trend during 2007–2011 in Gdansk Basin and Eastern Gotland Basin and 2007–2012 in the Bornholm Basin. From 2011 TP content in the Gdansk Basin and from 2012 in the Bornholm Basin started to gradually decrease until the unforeseen peak in 2017, while in Eastern Gotland Basin after 2011 there was no stable trend.

The DIP content was more stable than TP, but it also had an increasing trend in 2007–2017. Similarly to TP, the DIP content was the lowest in 2013 in Gdansk Basin and Bornholm Basin and in 2012 in Eastern Gotland Basin. The highest DIP content was observed in all three basins in 2016.

The DIN content trend was stable and almost constant in all three basins. In the Gdansk and Bornholm Basin, the highest DIN content was observed in 2017 while in Easter Gotland Basin in 2011. The lowest DIN vas achieved in Bornholm and Eastern Gotland basins in 2008 and 2012 in the Gdansk Basin.

### The relation between the analysed basins water quality and the nutrient loads from MWWTPs in Poland

**Fig. 7:**
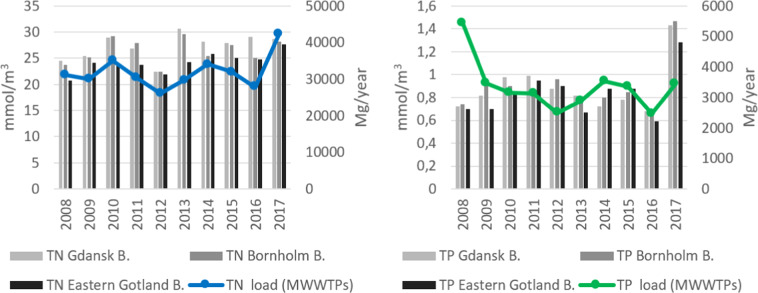
The relation between TP and TN content in the analysed basins and nutrient loads from MWWTPs discharges in 2008–2017

As an attempt to connect the excessive nutrient riverine loads discharged to the Polish EEZ the analysis of the relation was conducted using the annual loads reported by Polish MWWTPs and yearly concentrations of inorganic and total forms of N and P in the 3 analysed basins located in the Polish EEZ. The trends showing the relation variability for P are presented in Fig. 7.

Based on Fig. 7 it can be assumed that there is a visible relationship identified between the nutrients loads from MWWTPs and their concentration in the analysed basins. Especially, water TN concentration looks dependent from TN loads from MWWTPs in the whole analysed period, except 2013 and 2016. On the other hand, TP concentration seems to be less depended from the TP discharged loads however, in 2013–2017 a coherent trend was observed. The authors of the present study are aware that by having regard to the complexity of the eutrophication process it cannot be stated that only N and P loads from MWWTPs affect the actual water state in the analysed basins. However, the presented trends seem to be in accordance with the trophic state of coastal waters in the Polish EEZ during 2007–2017 what might suggest that municipal wastewater discharges have a significant impact on the water quality in terms of nutrient content and can lead to the changes in the water trophic state.

## Discussion

Besides the EU Member States also Russia and Belarus contribute to the Baltic Sea nutrient loads while they are not subject to EU legislation (Markowska and Zylicz 1999; Kiedrzyńska et al. 2014). It was estimated by the European Bank for Reconstruction and Development (EBRD) that wastewater treatment infrastructural projects approved by the Northern Dimension Environmental Partnership Support Fund (NDEPSF) may result in a reduction of TN by more than 7 600 Mg/year (7% of inflows from Russia and 9% from Belarus) and TP by 2 300 Mg/year (27% of inflows from Russia and 21% from Belarus) (European Court of Auditors 2016). Therefore, it would be a significant contribution to the required reduction of nutrients in the Baltic Sea.

The conducted research covered the actual wastewater nutrient loads impact from Poland on the water state of the Gdansk, Bornholm and Eastern Gotland Basins. However, the mentioned above water bodies are subject to nutrient loads from other countries such as the eastern part of Germany and Denmark (Bornholm), putting additional pressure on the Bornholm Basins. Gdansk Basin waters are also at risk of nutrient loads from Russia including the second-largest Russian agglomeration—the city of Kaliningrad. The Eastern Gotland Basin water quality is only partly shaped by the pollution loads from Poland due to its location in the centre of the Baltic Proper, which is considered mainly as transnational waters sensitive for nutrients especially from Poland, Russia, Lithuania and Latvia (Romanova and Preisner 2011).

The present study was based on actual wastewater discharge from annual reports which are the most accurate available data from Poland. The total nutrients flow used by HELCOM are in many cases different from the national statistics which might be caused by used assumption methodology (HELCOM 2017). Especially, diffuse nutrient sources are very dependent on assumptions and used models for the nutrient flow assessment from each specific catchment (Kay et al. 2012). Knowledge about the individual properties of catchment parts, seasonality, current agricultural practices trends is very valuable for estimations of diffuse nutrient pollution (Collins et al. 2018; Jetoo 2018).

The nutrient export to the Baltic Sea has been analysed by scientists in many Baltic region countries. A recent study from Finland (Räike et al. 2019) examined the trends in TN and TP concentrations and flow-normalised nutrients export into the Baltic Sea by all Finnish rivers in 1995–2016 in order to assess whether the Finnish nutrient reduction targets will be fulfilled by the 2021 deadline. Their results show that in Finnish rivers, TP concentrations correlate more closely with the rivers flow than TN concentrations. As Raike et al. noticed a positive correlation regarding nutrient content and river flow suggest an important role of diffuse sources in shaping the nutrient loads, which are determined by rainwater amount (Rankinen et al. 2019). On the contrary, if the correlation is negative, the nutrient load is more dependent on point sources (Räike et al. 2019). Regarding the Polish impact on the Baltic Sea, there is a similar situation with the current trends in the changes in riverine or direct nutrient sources. A decreasing trend was is observed in point sources nutrient loads since the previous development of advanced ENBR technologies in Polish MWWTPs, especially in terms of TP load.

It needs to be underlined that Poland and other Member States that joined EU in 2004 successfully negotiated a transitional period for compliance with the EU Directives which for Poland ended on 31^st^ December 2015 (Piasecki 2019), which means that the effects of all upgrades in the wastewater treatment sector are delayed compared to the older EU Member States (Biswas et al. 2018; Desmit et al. 2018). Similar to Finland, Polish TN riverine load is not decreasing despite the implementation of EU nutrient-related policies (Rankinen et al. 2016). Moreover, as the results of the present study show, there are unfavourable trends identified regarding the Baltic Sea coastal waters in the Polish EEZ in terms of total and inorganic nutrients content. This suggests that a wider scope is necessary to identify efficient wastewater and agricultural oriented policy means for further eutrophication mitigation in the Baltic Sea region.

## Conclusions

The Polish participation in the Baltic Sea pollution with biogenic compounds is the highest in the region. Although, in the recent years Poland has undertaken major measures in the field of counteracting eutrophication of the Baltic Sea by the implementation of among others the UWWTD and HELCOM recommendations, not many significant effects are observed regarding the actual coastal water quality. The inadequate impact of these activities on the actual state of the Polish coastal waters including the EEZ water bodies such as Gdansk Basin, Bornholm Basin and Eastern Gotland Basin is observed. However, downward trends for total nutrients input from Polish territory to the Baltic Sea have been achieved. Nevertheless, Poland remains the biggest polluter of the Baltic Sea with 6 838 Mg/year of TP load and 189 219 Mg/year of TN load in 2017. Regarding the nutrient loads from MWWTPs, it can be assumed that a decreasing trend is present for TP loads while a stable trend with temporary increases is visible in terms of TN loads in 2008-2018. This situation might be a result of the increasing share of households connected to MWWTPs by the sewage system. By the growing number of inhabitants connected to MWWTPs, it was possible to limit the number of leaking septic tanks and prevent groundwater pollution which is considered as an added value to the UWWTD implementation in Poland. Therefore, municipal effluent discharges by provoking certain biological processes can lead to changes in the trophic state of the Baltic Sea waters and the basins located in the Polish EEZ. However, other nutrient sources such as runoffs from agricultural areas, natural background sources and atmospheric deposition need to be investigated before making conclusive judgements about the policy implementation in the wastewater sector.
